# Factorial Structure of the EOCL-1 Scale to Assess Executive Functions

**DOI:** 10.3389/fpsyg.2021.585145

**Published:** 2021-05-31

**Authors:** Carlos Ramos-Galarza, Jorge Cruz-Cárdenas, Mónica Bolaños-Pasquel, Pamela Acosta-Rodas

**Affiliations:** ^1^Facultad de Psicología, Pontificia Universidad Católica del Ecuador, Quito, Ecuador; ^2^Centro de Investigación MIST y Carrera de Psicología, Universidad Tecnológica Indoamérica, Quito, Ecuador; ^3^Centro de Investigación ESTec, Universidad Tecnológica Indoamérica, Quito, Ecuador

**Keywords:** Alexander Luria, assessment, executive functions, EOCL-1, factorial analysis

## Abstract

The process of assessing executive functions through behavioral observation scales is still under theoretical and empirical construction. This article reports on the analysis of the factorial structure of the EOCL-1 scale that assesses executive functions, as proposed by the theory developed by Luria, which has not been previously considered in this type of evaluation. In this scale, the executive functions taken into account are error correction, internal behavioral and cognition regulatory language, limbic system conscious regulation, decision making, future consideration of consequences of actions, goal-directed behavior, inhibitory control of automatic responses, creation of new behavioral repertoires, and cognitive–behavioral activity verification. A variety of validity and reliability analyses were carried out, with the following results: (a) an adequate internal consistency level of executive functions between α = 0.70 and α = 0.83, (b) significant convergent validity with a scale that assesses frontal deficits between *r* = −0.07 and *r* = 0.28, and (c) the scale’s construct validity that proposes a model with an executive central factor comparative fit index (*CFI*) = 0.93, root mean square error of approximation (*RMSEA*) = 0.04 (LO.04 and HI.04), standardized root mean square residual (*SRMR*) = 0.04, and *x^2^_(__312__)_* = 789.29, *p* = 0.001. The findings are discussed based on previous literature reports and in terms of the benefits of using a scale to assess the proposed executive functions.

## Introduction

Alexander Luria developed one of the most interesting models for understanding the human brain’s functioning. This theory proposed that three functional units interact between different structures: the first is in charge of the regulation of wakefulness and tone; the second is in charge of the reception, process, and storage of information; the last unit is in charge of the programming, regulation, and verification of behavioral and cognitive activity (Luria, 1973, 1980, 1982).

The third functional unit is related to the work of the human prefrontal cortex (Derouesné, 2018), the structure responsible for the activation of the most developed mental activities of the nervous system, which are the executive functions (Cipolotti et al., 2020). These functions have been classically identified as the following mental abilities: (1) working memory, (2) inhibitory control, (3) emotional regulation, (4) monitoring, (5) planning, (6) organization, (7) initiative, and (8) cognitive flexibility (Téllez and Sánchez, 2016; Roche et al., 2020).

These eight executive functions have been described along with their theoretical development and in the different scales for their evaluation. However, this paper takes into consideration the functions of the frontal system described in Luria’s theory (Luris 1973, 1980): error correction, internal behavioral and cognition regulatory language, limbic system conscious regulation, decision making, future consideration of consequences of actions, goal-directed behavior, inhibitory control of automatic responses, creation of new behavioral repertories, and cognitive–behavioral activity verification, all functions that have not been included in the previous scales (Derouesné, 2018; Ramos-Galarza et al., 2019b).

Executive functions can be evaluated on three different levels: first, with specific experimental tasks that constitute neuropsychological tests developed with the aim of evaluating executive functions; second, with non-specific tasks that comprise tests created to evaluate different cognitive variables, such as intelligence, attention, and memory, which support the clinical evaluation of executive functions; and third, with scales or questionnaires based on daily life behavioral observation. It should be highlighted that the first two methods of evaluation have ecological validity limitations because the executive functions are not evaluated in real daily life. This explains why this paper proposes using the EOCL-1 scale (Appendix Table A1), which evaluates executive functions not previously considered based on the analysis of a subject’s daily situations (Damasio, 1994; García-Gómez, 2015; Pérez-Salas et al., 2016; Ramos-Galarza et al., 2019b).

The relevance of the assessment of executive functions through the application of questionnaires or scales lies in the contribution of daily life behaviors to the analysis. These types of tests have been found to have adequate ecological validity and reliability in evaluating these mental abilities in real contexts, which goes further than the artificial environment that a neuropsychological consulting room offers (Damasio, 1994; Pérez-Salas et al., 2016).

To determine the executive functions that were considered in previous scales, principal databases were reviewed, and scales such as Behavior Rating Inventory of Executive Functions (BRIEF), Prefrontal Symptoms Inventory (PSI), Executive Functioning Scale for Families (EFS-F), Scale of Executive Functions of the Cáceres group (EFECO), Adult Executive Functioning Inventory (ADEXI), Barkley Deficits in Executive Functioning Scale (BDEFS), and many others were identified as the main contributors in this research line. These scales focus their evaluation on the eight classically recognized executive functions in favor of behavioral regulation and metacognition. The revised scales are described in Table 1.

**TABLE 1:** Revision of the scales to assess executive functions.

**Investigation**	**Authors**	**Executive functions assessed**
Psychometric properties of the BRIEF scale to assess executive functions in a Spanish population	García et al., 2014	Inhibition
		Change
		Emotional control
		Working memory
		Planning
		Material organization
		Monitoring
		Initiative
Prefrontal Symptoms Inventory (PSI): ecological validity and convergence with neuropsychological measures	Pedrero et al., 2016	Emotional control
		Socially accepted behavior
		Task execution
Assessment of executive functions in children: Analysis and adaptation of tasks in a school context	Musso, 2009	Motor interference control
		Behavioral self-regulation
		Inhibition
BRIEF-A (short): Analysis of psychometric properties in a Spanish sample	Herreras, 2016	Change
		Working memory
		Initiative
		Planning
		Material organization
		Self-monitoring
		Inhibition
		Emotional control
Hierarchical structure analysis of behavior-rating inventory of executive functions–self-report in a sample of university students	Ramírez et al., 2017	Working memory
		Planning/organization
		Monitoring
		Change
		Inhibition
		Material organization
		Emotional control
		Task resolution
Psychometric characteristics of the Executive Functioning Scale for Families (EFS-F)	García et al., 2018	Impulsivity
		Hyperactivity
		Emotional control
		Working memory
		Planning capacity
		Organization capacity
		Flexibility
		Concentration capacity
		Focused attention
Executive functioning scale for schoolchildren: An analysis of psychometric properties	Korzeniowski and Ison et al., 2019	Attention control
		Inhibitory control
		Metacognition
		Organization
		Planning
		Cognitive flexibility
Evaluation of the skills of the prefrontal cortex: The EFECO II-VC And II-VR	Ramos-Galarza et al., 2018	Impulsive response control
		Cognitive flexibility
		Emotionally deliberated control
		Capacity to act initiatively
		Planning capacity
		Capacity to organize elements to solve a task
		Cognitive and monitoring capacity
		Working memory
		Verification
EFECO scale for assessing executive functions in self-report format	Ramos-Galarza et al., 2019c	Monitoring
		Cognitive flexibility
		Material organization
		Initiative
		Working memory
		Planning
		Emotional regulation
		Inhibitory control
Bifactor Modeling of the Behavior Rating Inventory of Executive Functions (BRIEF) in a Chilean Sample	Pérez-Salas et al., 2016	Inhibition
		Cognitive flexibility
		Emotional control
		Initiative
		Working memory
		Organization/planning
		Material organization
		Monitoring
Self-report measures of executive functioning as a determinant of academic performance in first-year students at a university of applied sciences	Baars et al., 2015	Planning
		Attention
		Self-control (inhibition)
		Monitoring
The role of executive functions in academic performance and behavior of university students	Ramos-Galarza et al., 2019a	Difficulties in working memory
		Difficulties in consciously controlled behavior and emotional regulation
		Difficulties in the organization of elements in order to solve tasks
		Difficulties in the conscious supervision of behavior
Multidomain self-report assessment of fronto-executive complaints in Spanish-speaking adults	Miranda et al., 2019	Executive attention
		Cognitive flexibility
		Inhibitory control
Reliability and validity of the Thinking Skills Inventory, a screening tool for cross-diagnostic skill deficits underlying youth behavioral challenges	Wang et al., 2019	Working memory
		Emotional regulation
		Cognitive flexibility
		
Developing and validating a big-store multiple errands test	Antoniak et al., 2019	Problem-solving
		Planning
		Monitoring
		Adaptation to novel situations
Psychometric properties of Persian version of the Barkley Deficits in Executive Functioning Scale–Children and Adolescents	Mashhadi et al., 2020	Time management
		Self-organization/problem-solving strategies
		Self-control/inhibition
		Self-motivation
		Emotional regulation
Assessment of everyday executive functioning using the BRIEF in children and adolescents treated for brain tumors	Roche et al., 2020	Inhibition, change, emotional control, behavioral regulation, initiative, working memory, planning/organization, materials organization, monitoring, metacognition, and general executive functions
Refinement and psychometric evaluation of the Executive Skills Questionnaire-Revised	Strait et al., 2019	Planning management, time management, materials organization, emotional regulation, and behavioral regulation
Adult Executive Functioning Inventory (ADEXI): Validity, reliability, and relation to ADHD	Holst and Thorell, 2018	Working memory and inhibitory control
Barkley Deficits in Executive Functioning Scale (BDEFS)	Barkley, 2011	Self-organization/problem solving, self-restraint, self-motivation, and self-regulation of emotion

As the results of the literature revision on the scales emerged, it became evident that the interest in assessing the executive functions classically recognized in the theories of Lezak (1995); Isquith and Gioia (2008), Baddeley (2012); Barkley (2014) did not consider certain executive functions, such as verification, error correction, and awakening state, which are key functions of the frontal lobe. In this context, it also became apparent that to consider the executive functions described in Luria’s theory, as part of the neuropsychological evaluation through daily life assessment, would be both enriching and innovating.

Thus, this investigation proposes using the EOCL-1 scale, which allows for the assessment of executive functions as described in the theory developed by Luria (Ramos-Galarza et al., 2019b), which assesses frontal lobe abilities that were not considered previously in scales or questionnaires, thus richly contributing to the executive functions’ instruments frame, as well as to its theoretical construction. Among the previous investigations, there is no published study about this scale and its psychometric properties; therefore, this article reports the results of the investigations that analyzed the factorial structure, internal consistency, and convergent validity of the EOCL-1 scale.

## Investigation Hypotheses

Based on Luria’s theoretical proposal, nine executive functions were identified that were not considered previously in the tests already developed: EF 1, Error correction; EF 2, Internal behavior and cognition regulatory language; EF 3, Limbic system conscious regulation; EF 4, Decision making; EF 5, Future consideration of consequences of actions; EF 6, Goal-directed behavior; EF 7, Inhibitory control of automatic responses; EF 8, Creation of new behavioral repertoires; and EF 9, Cognitive–behavioral activity verification. The content of the 27 items of the EOCL-1 scale were proposed by the research team after a deep analysis of Luria’s theory (Luria, 1973, 1980, 1982), together with the team’s clinical experience of treating people with frontal lobe impairments. This allowed comprehension of the differences between the pathological and normal states of executive functioning. The authors propose the following hypotheses in relation to EOCL-1 reliability:

H_1_. The nine executive functions assessed with the EOCL-1 scale will show adequate psychometric properties in terms of internal consistency.

Most of the scales developed to evaluate the executive functions focus on the deficits in these mental abilities, which negatively influences self-reporting. One of the scales that mainly focus on deficits is the Prefrontal Symptoms Inventory (Ruiz-Sánchez de León et al., 2012), an instrument that assesses the executive functions inversely to the direction that the EOCL-1 promotes. Based on that description, the next hypothesis proposes a convergent validity based on an inverse relationship between the Prefrontal Symptoms Inventory and the EOCL-1 scale, since the high scores in the executive function deficits of the people evaluated will correlate with low scores in the EOCL-1 scale.

H_2_. The EOCL-1 scale will present a statistically significant convergent validity based on an inversely proportional correlation to the Prefrontal Symptoms Inventory because the EOCL-1 scale assesses the ability of executive functions, while the Prefrontal Symptoms Inventory measures frontal cortex structure deficits.

**FIGURE 1:**
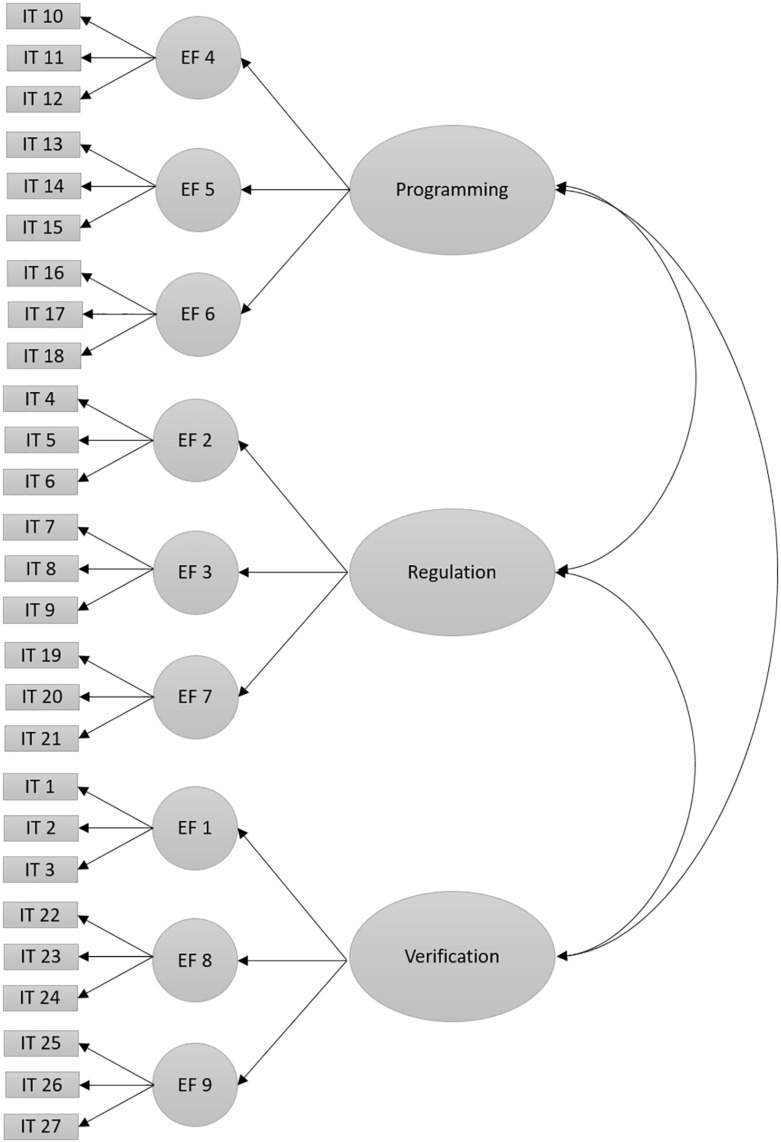
Three-factor model of second order of the EOCL-1 scale (Model 1). Note. EF 1, error correction; EF 2, internal behavioral and cognition regulatory language; EF 3, limbic system conscious regulation; EF 4, decision making; EF 5, future consideration of the consequences of actions; EF 6, goal-directed behavior; EF 7, inhibitory control of automatic responses; EF 8, creation of new behavioral repertoires; EF 9, cognitive–behavioral activity verification.

Luria’s theory (Luria, 1973, 1980, 1982) proposes that the frontal brain system, the neuroanatomic structure responsible for executive functions, works on three levels: programming, verification, and regulation, and, as such, it allows humans to consciously regulate their behavior and cognition (Ramos-Galarza et al., 2019b). Based on this theoretical postulate, the next hypothesis about the scale’s factorial structure is proposed:

H_3_. The EOCL-1 scale will show an adequate goodness-of-fit of the confirmatory factor model because it assesses nine executive functions organized in the three factors of second order proposed by Luria’s theory: programming, regulation, and verification (model 1, Figure 1).

**FIGURE 2:**
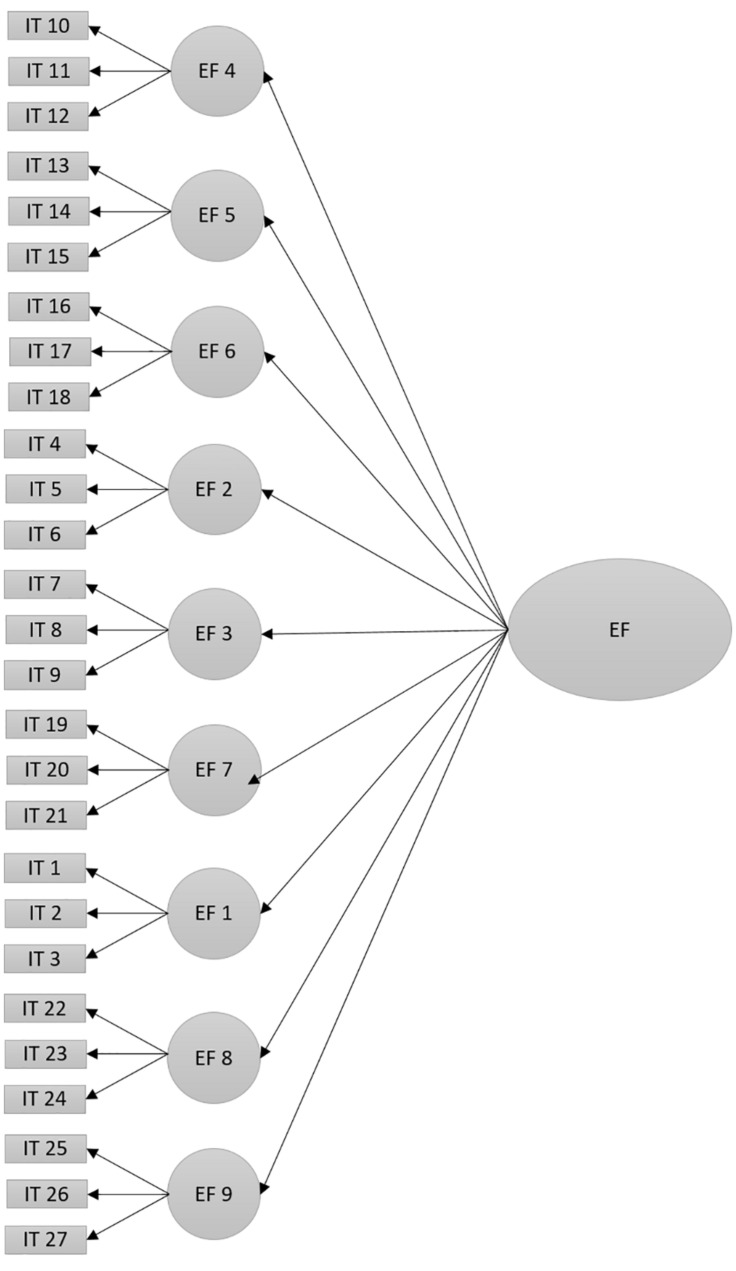
Factorial model of second order with one executive central factor of the scale EOCL-1 (Model 2). Acronyms are the same as in Figure 1.

The executive function models that have been developed include those based on one executive central factor (Norman and Shallice, 1986; Barkley, 1997), as well as multiple factors (Gioia et al., 2002; García-Gómez, 2015; Ramírez et al., 2017; Kamradt et al., 2019), and have proposed the theoretical organization of these mental abilities. It is important to highlight that there are no conclusive results as yet, and, based on this description, the following two hypotheses are proposed, one based on a central executive factor and the other based on multiple factors:

H_4_. The EOCL-1 scale will present an adequate goodness-of-fit of the confirmatory factor analysis of the model of second order organized around one central factor (model 2, Figure 2).

**FIGURE 3:**
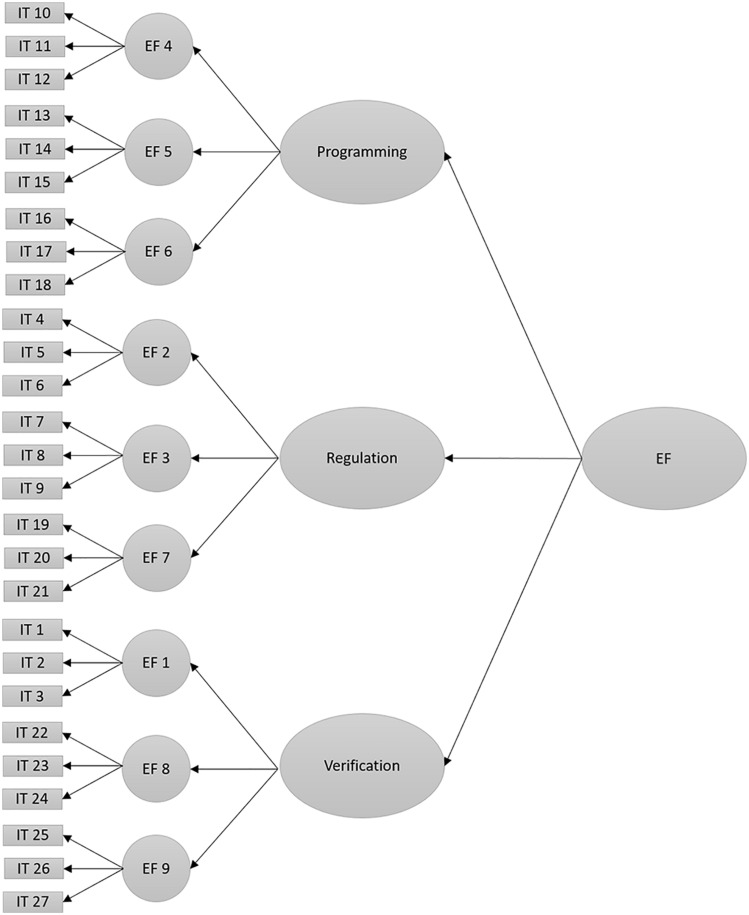
Factorial model of one factor of third order and three factors of second order of the scale EOCL-1 (Model 3). Note: Acronyms are the same as in Figure 1.

H_5_. The EOCL-1 scale will show an adequate goodness-of-fit of the confirmatory factor analysis in the model of one executive factor of third order and the three factors of second order proposed in the theory developed by Luria: programming, regulation, and verification (model 3, Figure 3).

The authors consider that based on Luria’s theory, there will be a model with a higher heuristic to comprehend humans’ executive functioning, and the following hypothesis proposes the model that will have the best adjustment level:

H_6_. The model of the EOCL-1 scale that rests on one factor of third order and three factors of second order (programming, regulation, and verification), based on Luria’s theory (model 3, Figure 3), will show a better statistically significant goodness-of-fit when compared with the rest of the models proposed.

## Method

### Sample

Multiple-level sampling was done, first, by a selection of zones from the urban-metropolitan district of Quito-Ecuador, next, of 134 neighborhoods from this district, and, finally, by the random choice of 771 participants within each neighborhood. The number of participants allows counting with an adequate statistical power for this research. The scale consists of 27 items and therefore is equivalent to 28.55 people for each proposed item.

The application of the questionnaires took place between September 2019 and January 2020. The authors and clinical psychologists trained in their implementation applied the instruments. The first step of the data analysis was obtaining the main descriptive statistics of the sample and comparing them with those of the general city’s population. The average age of the participants was 39.86 years (*SD* = 15.47), which is very close to the average age of Quito’s population, which is 39 years old (National Institute of Statistics and Censuses [INEC], 2010).

The inclusion criteria were as follows: (a) living within Quito-Ecuador’s urban metropolitan district, (b) not presenting a disability, (c) not having a record of psychopathological diagnosis, (d) having the capacity to understand and answer the items presented, (e) signing the informed consent of voluntary participation, and (f) being at least 18 years old. The exclusion criteria were as follows: (a) having a record of psychopathological diagnosis or any psychopharmacological consumption, (b) having a disability, and (c) showing an unwillingness to participate in the research.

Table 2 presents other statistics of interest. As can be seen, the percentage of women surveyed (50.5%) was very close to the percentage of men surveyed (49.5%). Additionally, the educational level most frequently reached by the respondents was secondary school (48.5%), which is precisely the predominant educational level of Quito’s adult population (National Institute of Statistics and Censuses [INEC], 2010, 2013). Based on these data, it can be concluded that the sample extracted is an adequate representation of Quito’s population.

**TABLE 2:** Sample characteristics (*N* = 771).

**Variable**	**(%)**
**Gender**	
Women	50.50%
Men	49.50%
**Age (years)**	
Younger than 26	23.60%
26–45	41.60%
46 years and above	34.80%
**Educational level attained**	
Incomplete secondary level or less	14.50%
Complete secondary level	48.50%
Incomplete university level	18.30%
Undergraduate/graduate university level	18.70%
**Monthly family income**	
Less than $600	39.30%
601–$1,400	40.50%
1,401–$2,200	13.00%
2,201 or more	7.30%

### Measurements

In this investigation, two instruments were applied: the EOCL-1 scale (Ramos-Galarza et al., 2019b) and the Prefrontal Symptoms Inventory (Ruiz-Sánchez de León et al., 2012). The EOCL-1 scale was developed based on Luria’s theory, in which executive functions not considered in the scales previously developed were included for assessment. The reason for the absence of these executive functions could be because the scales developed so far were created by the classic authors in the field. However, according to Luria’s proposed model, mental abilities of the frontal cortex that are used in daily life behavior are included, as follows: EF1, error correction; EF2, internal behavioral and cognition regulatory language; EF3, limbic system conscious regulation; EF4, decision making; EF5, future consideration of consequences of actions; EF6, goal-directed behavior; EF7, inhibitory control of automatic responses; EF8, creation of new behavioral repertoires; and EF9, cognitive–behavioral activity verification. One of the benefits of this scale is the directionality in the redaction of its composing items, since it assesses the functions positively, which allows for analyzing the ability of executive functioning in the diverse contexts in which an adult develops. It is also a free-use scale (Table A1).

The second scale used to evaluate EOCL-1 converged validity, the Prefrontal Symptoms Inventory, is a questionnaire that assesses the signs and symptoms of prefrontal cortex impairments from a behavioral perspective. Therefore, when applying these two scales, convergent validity is expected, which will show an inversely proportional relationship among the executive function abilities assessed with the EOCL-1 scale and their impairment, as measured by the Prefrontal Symptoms Inventory.

The Prefrontal Symptoms Inventory has a negative narrative in that it focuses on deficits in executive functioning. The following factors are evaluated with this instrument: (a) social–behavioral problems, (b) behavioral–control problems, and (c) emotional–control problems. Adequate values of validity and reliability have been reported using this instrument (Ruiz-Sánchez de León et al., 2012; Table A2).

### Procedure

This study started with the approbation from the Ethical Committee of the Universidad Indoamérica del Ecuador. The application of the instruments was done at each participant’s house from the randomized assignation. In every case, an informed consent of volunteer and anonymous participation was obtained. Throughout the study, the participants’ physical and psychological integrity were safeguarded. Once the instruments were applied, the data were registered and organized in the databases in preparation for the statistical analysis.

### Statistical Analysis

The statistical procedures of central tendency and dispersion were applied to evaluate the descriptive data. To analyze the reliability of each subscale, Cronbach’s alpha and McDonald’s Omega procedures were applied. To analyze convergent validity, Pearson’s correlation procedure was applied. Finally, a confirmatory factor analysis was conducted to evaluate the construct validity of the model hypothesized. The values to determine an acceptable index of goodness-of-fit in the factorial analysis were comparative fit index (*CFI*) ≥ 0.95, root mean square error of approximation (*RMSEA*) < 0.06, and standardized root mean square residual (*SRMR*) ≤ 0.08 (Schreiber et al., 2005).

These analyses were carried out in the statistical package SPSS v. 25 and Amos v. 23.

### The Study Scenario

This study was conducted in the capital of Ecuador, the city of Quito. Ecuador is a middle-income developing country located in South America. Ecuador has an approximate population of 17 million inhabitants, while the city of Quito has, in its metropolitan area, a population close to 3 million inhabitants. Ecuador’s economic system is based on the private property model, and its official currency is the US dollar (National Institute of Educational Evaluation, 2019). Thus, the following presented results could be useful not solely in the context in which this research took place but also in other contexts that share similar described characteristics.

## Results

### Descriptive Data

Table 3 presents the descriptive data values obtained in the nine executive functions evaluated with the EOCL-1 scale.

**TABLE 3:** Scale factors’ descriptive statistics.

**Factor**	***M***	***SD***	**Mn**	**Mx**	**As**	**Kr**
EF1	30.36	3.56	15	35	−0.61	0.23
EF2	20.33	3.61	5	25	−0.93	1.21
EF3	19.52	3.61	5	25	−0.63	0.50
EF4	21.66	2.67	12	25	−0.65	−0.31
EF5	20.54	3.18	6	25	−0.61	0.35
EF6	17.30	2.44	4	23	−1.04	1.91
EF7	20.79	3.16	7	25	−0.73	0.57
EF8	21.43	2.66	11	25	−0.86	0.91
EF9	17.27	2.32	8	20	−0.76	0.47

*M, mean; SD, standard deviation; Mn, minimum; Mx, maximum; As, asymmetry; Kr, kurtosis.*

### Internal Consistency—First Hypothesis

To analyze the internal consistency of the executive functions, Cronbach’s alpha and McDonald’s Omega procedures were applied, as follows. The possible variability in the ordinal measurement of each item was controlled, and the internal consistency of the applied scale can be highly understood (Elosua and Zumbo, 2008; Lozano et al., 2008). The found values in this analysis were EF1, α = 0.82 and ω = 0.77; EF2, α = 0.83 and ω = 0.85; EF3, α = 0.83 and ω = 0.84; EF4, α = 0.77 and ω = 0.72; EF5, α = 0.75 and ω = 0.72; EF6, α = 0.73 and ω = 0.78; EF7, α = 0.72 and ω = 0.69; EF8, α = 0.76 and ω = 0.74; EF9, α = 0.70 and ω = 0.68.

The values found provide empirical evidence in favor of the first hypothesis proposed, since the levels of internal consistency are in the range of acceptable reliability.

### Convergent Validity—Second Hypothesis

For the analysis of convergent validity, the Prefrontal Symptoms Inventory was applied (Ruiz-Sánchez de León et al., 2012). The following scales were used: (a) social–behavioral problems, (b) behavioral–control problems, and (c) emotional–control problems (Table 4 shows the descriptive analysis).

**TABLE 4:** Descriptive data of the Prefrontal Symptoms Scale.

	**Min**	**Max**	**Mean**	***SD***
Social–behavioral problems	4.00	20.00	7.25	3.39
Behavioral–control problems	9.00	45.00	20.33	7.05
Emotional–control problems	3.00	15.00	7.08	2.70

Subsequently, the analysis of the correlation between Luria’s EOCL-1 scale and the Prefrontal Symptoms Inventory resulted in a statistically significant inversely proportional correlation between the variables (Table 5).

**TABLE 5:** Correlation between EOCL-1 and prefrontal symptoms.

**Factors**	**EF1**	**EF2**	**EF3**	**EF4**	**EF5**	**EF6**	**EF7**	**EF8**	**EF9**
A	−0.23**	−0.07**	−0.16**	−0.18**	−0.14**	−0.15**	−0.14**	−0.23**	−0.16**
B	−0.19**	−0.09**	−0.13**	−0.20**	−0.22**	−0.21**	−0.24**	−0.23**	−0.18**
C	−0.15**	−0.12**	−0.22**	−0.18**	−0.26**	−0.15**	−0.28**	−0.17**	−0.08**

** Significance at 0.001. Key of factors. A, Social–behavioral problems; B, Behavioral–control problems; C, Emotional–control problems.

The results found in the correlational analysis provide empirical evidence in favor of the second hypothesis proposed; since the correlation is inversely proportional, it is proposed that the EOCL-1 scale has a positive directionality in favor of the executive functions, while the Prefrontal Symptoms Inventory is aimed at assessing the deficits in executive functioning.

### Confirmatory Factor Analysis: Model of Second Order in Three Executive Abilities—Third Hypothesis

A model of second-order factors was proposed following Luria’s theory, in which the three levels of the frontal lobe brain system were considered, i.e., the programming, regulation, and verification of cognitive and behavior activity. The model hypothesized was composed of three principal factors: programming (comprises decision making, future consideration of consequences of actions, and goal-directed behavior), regulation (comprises internal behavioral and cognition regulatory language, conscious regulation of the limbic system, and control of automatic responses), and verification (comprises error correction, creation of new behavioral repertoires, and cognitive–behavioral activity verification) (Figure 1 shows the hypothetical model, and Table 6 shows the regression weights). The results provide empirical evidence in favor of the third hypothesis proposed, since the goodness-of-fit parameters found contribute to the model proposed: *CFI* = 0.93, *RMSEA* = 0.04 (LO.04 and HI.05), *SRMR* = 0.04, and *x^2^_(__312__)_* = 789.29, *p* = 0.001.

**TABLE 6:** First model’s regression weights.

	**Model 1**				
**Regressions**			**Estimate**	**SE**	**CR**
EF 4	<—	PROGRAMMING	1.27	0.14	8.86**
EF 5	<—	PROGRAMMING	1.43	0.18	7.79**
EF 6	<—	PROGRAMMING	1.00		
EF 3	<—	REGULATION	0.90	0.10	9.44**
EF 7	<—	REGULATION	1.00		
EF 2	<—	REGULATION	0.82	0.09	8.85**
EF 1	<—	VERIFICATION	0.88	0.09	10.12**
EF 8	<—	VERIFICATION	0.87	0.09	9.85**
EF 10	<—	VERIFICATION	1.00		
IT 1	<—	EF 1	1.00		
IT 2	<—	EF 1	1.20	0.08	14.73**
IT 3	<—	EF 1	1.09	0.08	14.37**
IT 4	<—	EF 2	1.00		
IT 5	<—	EF 2	1.01	0.05	22.03**
IT 6	<—	EF 2	1.04	0.05	21.64**
IT 7	<—	EF 3	1.00		
IT 8	<—	EF 3	1.09	0.05	20.76**
IT 9	<—	EF 3	1.07	0.05	20.06**
IT 10	<—	EF 4	1.00		
IT 11	<—	EF 4	0.91	0.06	15.46**
IT 12	<—	EF 4	0.97	0.06	17.15**
IT 13	<—	EF 5	1.00		
IT 14	<—	EF 5	0.84	0.08	10.67**
IT 15	<—	EF 5	0.96	0.09	10.87**
IT 16	<—	EF 6	1.00		
IT 17	<—	EF 6	0.92	0.07	14.04**
IT 18	<—	EF 6	0.96	0.07	13.13**
IT 19	<—	EF 7	1.00		
IT 20	<—	EF 7	1.01	0.08	12.70**
IT 21	<—	EF 7	0.87	0.07	11.65**
IT 22	<—	EF 8	1.00		
IT 23	<—	EF 8	0.90	0.07	12.58**
IT 24	<—	EF 8	0.94	0.07	13.52**
IT 25	<—	EF 9	1.00		
IT 26	<—	EF 9	0.96	0.08	11.75**
IT 27	<—	EF 9	0.76	0.07	11.25**

**p < 0.001.

### Confirmatory Factor Analysis: Model of Second Order Organized in a Central Executive Factor—Fourth Hypothesis

Furthermore, a model of a unique factor of executive functions was analyzed, which found a goodness-of-fit of *CFI* = 0.93, *RMSEA* = 0.04 (LO.04 and HI.05), *SRMR* = 0.04, and *x^2^_(__315__)_* = 791.05, *p* = 0.001. These results provide empirical evidence in favor of the proposed hypothesis (Figure 2 shows the hypothetical model, and Table 7 shows the regression weights).

**TABLE 7:** Second model’s regression weights.

	**Model 2**				
**Regressions**			**Estimate**	**SE**	**CR**
EF 4	<—	EF	0.88	0.09	10.22**
EF 3	<—	EF	0.93	0.10	9.20**
EF 1	<—	EF	0.88	0.09	10.12**
EF 2	<—	EF	0.86	0.10	8.75**
EF 5	<—	EF	1.01	0.12	8.70**
EF 6	<—	EF	0.69	0.08	8.28**
EF 7	<—	EF	1.03	0.11	9.57**
EF 9	<—	EF	1.00		
EF 8	<—	EF	0.87	0.09	9.83**
IT 1	<—	EF 1	1.00		
IT 2	<—	EF 1	1.20	0.08	14.73**
IT 3	<—	EF 1	1.10	0.08	14.36**
IT 4	<—	EF 2	1.00		
IT 5	<—	EF 2	1.01	0.05	22.04**
IT 6	<—	EF 2	1.04	0.05	21.65**
IT 7	<—	EF 3	1.00		
IT 8	<—	EF 3	1.09	0.05	20.74**
IT 9	<—	EF 3	1.07	0.05	20.05**
IT 10	<—	EF 4	1.00		
IT 11	<—	EF 4	0.91	0.06	15.46**
IT 12	<—	EF 4	0.97	0.06	17.12**
IT 13	<—	EF 5	1.00		
IT 14	<—	EF 5	0.84	0.08	10.69**
IT 15	<—	EF 5	0.96	0.08	10.89**
IT 16	<—	EF 6	1.00		
IT 17	<—	EF 6	0.92	0.07	14.03**
IT 18	<—	EF 6	0.96	0.07	13.13**
IT 19	<—	EF 7	1.00		
IT 20	<—	EF 7	0.99	0.08	12.70**
IT 21	<—	EF 7	0.86	0.07	11.65**
IT 22	<—	EF 8	1.00		
IT 23	<—	EF 8	0.90	0.07	12.56**
IT 24	<—	EF 8	0.94	0.07	13.53**
IT 25	<—	EF 9	1.00		
IT 26	<—	EF 9	0.96	0.08	11.75**
IT 27	<—	EF 9	0.76	0.07	11.23**

**p < 0.001.

### Confirmatory Factor Analysis: Model of the Third Factor in One Executive Central Factor and Three Executive Factors of Second Order—Fifth Hypothesis

Next, a model of a unique factor of executive functions was analyzed, which found a goodness-of-fit of *CFI* = 0.93, *RMSEA* = 0.04 (LO.04 and HI.04), *SRMR* = 0.04, and *x^2^_(__312__)_* = 789.29, *p* = 0.001. These values provide evidence in favor of the proposed hypothesis (Figure 3 shows the hypothetical model, and Table 8 shows the regression weights).

**TABLE 8:** Third model’s regression weights.

	**Model 3**				
**Regressions**			**Estimate**	**S.E.**	**C.R.**
PROGRAMMING	<—	EF	0.72	0.09	7.96**
REGULATION	<—	EF	1.01	0.11	9.14**
VERIFICATION	<—	EF	1.00		
EF 4	<—	PROGRAMMING	1.27	0.14	8.86**
EF 5	<—	PROGRAMMING	1.43	0.18	7.79**
EF 6	<—	PROGRAMMING	1.00		
EF 3	<—	REGULATION	0.90	0.10	9.44**
EF 7	<—	REGULATION	1.00		
EF 2	<—	REGULATION	0.80	0.09	8.85**
EF 1	<—	VERIFICATION	0.88	0.09	10.13**
EF 8	<—	VERIFICATION	0.87	0.09	9.85**
EF 9	<—	VERIFICATION	1.00		
IT 1	<—	EF 1	1.00		
IT 2	<—	EF 1	1.20	0.08	14.73**
IT 3	<—	EF 1	1.09	0.08	14.37**
IT 4	<—	EF 2	1.00		
IT 5	<—	EF 2	1.01	0.05	22.03**
IT 6	<—	EF 2	1.04	0.05	21.64**
IT 7	<—	EF 3	1.00		
IT 8	<—	EF 3	1.09	0.05	20.76**
IT 9	<—	EF 3	1.07	0.05	20.06**
IT 10	<—	EF 4	1.00		
IT 11	<—	EF 4	0.91	0.06	15.46**
IT 12	<—	EF 4	0.97	0.06	17.15**
IT 13	<—	EF 5	1.00		
IT 14	<—	EF 5	0.84	0.08	10.67**
IT 15	<—	EF 5	0.96	0.09	10.87**
IT 16	<—	EF 6	1.00		
IT 17	<—	EF 6	0.92	0.07	14.04**
IT 18	<—	EF 6	0.96	0.07	13.13**
IT 19	<—	EF 7	1.00		
IT 20	<—	EF 7	1.00	0.08	12.70**
IT 21	<—	EF 7	0.87	0.07	11.65**
IT 22	<—	EF 8	1.00		
IT 23	<—	EF 8	0.90	0.07	12.58**
IT 24	<—	EF 8	0.94	0.07	13.52**
IT 25	<—	EF 9	1.00		
IT 26	<—	EF 9	0.96	0.08	11.75**
IT 27	<—	EF 9	0.76	0.07	11.25**

**p < 0.001.

### Comparison of the Different Factorial Models—Sixth Hypothesis

To analyze the model that presented the best goodness-of-fit, comparing the three models proposed, it was necessary to consider each model’s adjustment values, as well as their goodness-of-fit values. As a result, it was found that the three models obtained similar values, which is consistent with Luria’s theory. However, the model that proposes an executive central factor shows a slightly better adjustment, and because of this, the empirical evidence does not provide conclusive evidence to accept the sixth hypothesis of this investigation.

### Invariance Analysis

The invariance analysis was carried out for the model that presented the better goodness-of-fit, following the steps recommended by Byrne (2016). As a first step, the configurational or no restriction model (Model 1) was estimated; the same shape of model was applied to both groups, but with no equivalence restriction among the parameters. After this, Model 2 was estimated, in which all the factorial loading indicators were the same among the groups. Finally, in Model 3, in addition to the Model 2 restrictions, it was imposed that the structural coefficient of the paths remains the same throughout the groups. The base model for the comparison was Model 1.

For the comparisons between the models, the criteria of Cheung and Rensvold (2002); Chen (2007) were followed, and the chosen comparison indexes were the variations of *CFI* and *RMSEA* (Δ*CFI* and Δ*RMSEA*), since *CFI* and *RMSEA* are less sensitive to the sample size than the chi-square index (χ^2^). Additionally, and as per the recommendations of the cited authors, the cutting points to understudy the invariance were fixed in a maximum change of absolute value of *CFI* of.010, together with a maximum change in absolute value of *RMSEA* of.015.

As shown in Table 9, the values of Δ*CFI* and Δ*RMSEA*, as a result of the comparison between Models 2 and 3 against Model 1, all the cases obtained less values than the cutting selected points. These results support the existence of the model’s invariance, meaning that the proposed model is equivalent for both genders, men and women.

**TABLE 9:** Statistical adjustment for invariance analysis.

**Model**	**Comparison**	**Chi square (*X*^2^)**	**df**	**RMSEA**	**ΔRMSEA**	**CFI**	**ΔCFI**
Model 1: No restricted or configurational		1,290.29	624	0.037		0.900	
Model 2: Equal factorial loading	2 vs. 1	1,319.30	642	0.037	0	0.899	-0.001
Model 3: Factorial loading and structural equal coefficients	3 vs. 1	1,344.67	650	0.037	0	0.896	-0.004

*CFI, comparative fit index; RMSEA, root mean square error of approximation.*

## Discussion

This article reports an analysis of the factorial structure of the EOCL-1 scale to assess executive functions based on Luria’s mental abilities theory. The aim of this investigation is to generate a significant contribution to the executive function research line, with the proposal of a measurement instrument to evaluate a variety of these mental abilities, which were not considered previously in other scales that assess daily life executive functions (Ramos-Galarza et al., 2019b).

The executive functions that are proposed in this investigation, within the scale’s factorial structure and after a revision of the previous scales, are a novel contribution to this research line. These functions include the executive frontal abilities of error correction, internal behavioral and cognition regulatory language, limbic system conscious regulation, decision making, future consideration of consequences of actions, goal-directed behavior, inhibitory control of automatic responses, creation of new behavioral repertoires, and cognitive–behavioral activity verification.

In the analysis of the EOCL-1 scale factorial structure, it was found that the three proposed models showed an adequate adjustment; however, the empirical evidence is not sufficient to accept one model as more valid than another, but the models proposed allow us to understand the executive functions’ organization from the proposed theory. The model of the unique factor could have a better adjustment, although future investigations are necessary to analyze the proposed hypothesis with greater depth.

This investigation contributes to the development of two new aspects within the executive function line of research. The first relates to theoretical construction, since, at the moment, there is not an absolute consensus as to how many executive functions can be generated by the prefrontal cortex. The second aspect pertains to the instruments that measure these mental abilities, since many of the observational scales for executive functions have arisen from the theories of classic neuropsychology and, as explained in this work, are missing the relevant mental abilities proposed by Alexander Luria.

Regarding the content of this instrument, it is important to highlight the purpose of the proposed items, since they are meant to evaluate the positive aspects of executive abilities, with the benefit of evaluating these in the different contexts in which human beings develop, such as in work, education, and social environments. Thus, the confusion that is often generated in the inversely proportional correlations could be avoided if the evaluation were allowed to focus naturally on people’s executive abilities, without the bias of evaluating their deficits.

In reference to the convergent validity, the contribution of this scale in favor of the assessment of executive functions is evident. Since an inverse relationship was found with the scale that evaluates the deficit of frontal functions, interest in developing different research lines around executive functions in other contexts naturally arises. Furthermore, the possibility is opened for applying the proposed scale in a clinical environment to study people with neuropsychological alterations and to look closer into human cognition and behavior in other areas of interest.

The inversely proportional correlation found in the convergent validity analysis is in concordance with our hypothesis assumptions, since the low punctuation in the EOCL-1 scale indicates a low level of executive functioning, inversely to the higher punctuations in the scale that assesses frontal lobe deficits. Therefore, the EOCL-1 scale is sensitive in detecting executive functioning deficits, as well as in assessing the typical performance of these mental abilities.

The results found and presented in this article are in accordance with previous literature, as well as with the proposals of the following scales: BRIEF (Roche et al., 2020), Executive Skills Questionnaire (Strait et al., 2019), ADEXI (Holst and Thorell, 2018), BDEFS (Barkley, 2011), the BDEFS–Children and Adolescents (Mashhadi et al., 2020), and the Multidomain Self-Report Assessment of Fronto-Executive Complaints in Spanish-Speaking Adults (Miranda et al., 2019), which have described the psychometric properties in favor of evaluating the executive functions related to behavioral conscious regulation, cognition, and emotion.

In relation to the impact of the scale proposed, it is important to highlight its free use in the clinical context as a screening tool to assess executive functions from the ecological perspective of daily life. Moreover, when considering the measurement of executive functions not previously considered in these types of observational scales of behavior, the possibility emerges of executing new investigations in which the impact of the executive functions is considered in the novel contexts in which individuals develop in educational, family, social, and personal arenas.

In addition, when considering novel executive functions and an assessment proposal, this study makes a significant contribution to the line of research on executive functions, which is still under theoretical construction and in which the methods for assessing these mental abilities are still in development. In this way, the proposed research has an impact in opening new lines of research in executive functions not previously considered through this method of evaluation, such as error correction, future consideration of consequences of actions, decision making, and other functions included in the scale.

Another theoretical implication that emerges from this research and from a broad theoretical review of the scales developed to evaluate executive functions lies in the use and foundation of classical theoretical models (Musso, 2009; Barkley, 2011; Baars et al., 2015; Herreras, 2016; García et al., 2018; Holst and Thorell, 2018; Antoniak et al., 2019; Korzeniowski and Ison, 2019; Strait et al., 2019; Wang et al., 2019; Mashhadi et al., 2020; Roche et al., 2020). This research attempts to go beyond the established models by proposing such an assessment from the perspective of the behavioral observation of novel executive functions. Moreover, with this contribution, new theoretical models can be created that allow us to further understand the complexity that executive functions imply and to continue contributing to the study of the relations of these mental abilities of the frontal lobe and the processes of intervention in individuals who are suffering from acquired brain damage.

According to the influence of sociodemographic variables in the tested model, it was found that the gender variable during the invariance analysis had no influence in the EOCL-1 model, which explains why this construct has a similar behavior when applied to men and women. Finally, the limitation that must be declared about this investigation relates to the place where the sample was taken, in a specific city from Latin America, which has to be taken into account when generalizing the results. However, it is this very point on which future investigation lies, which is the interest in analyzing the psychometric behavior of the EOCL-1 scale in the different geographical contexts and cultures in which human beings develop in order to gain a deeper comprehension of the role that the executive functions play in daily life performance.

## Data Availability Statement

The raw data supporting the conclusions of this article will be made available by the authors, without undue reservation.

## Ethics Statement

The studies involving human participants were reviewed and approved by the Comité de Ética de Investigación con Seres Humanos Universidad Tecnológica Indoamérica. The patients/participants provided their written informed consent to participate in this study.

## Author Contributions

CR-G contributed to the conceptualization, investigated the data, carried out the formal analysis and project administration, wrote the original draft, and reviewed and edited the manuscript. JC-C investigated the data, carried out the formal analysis, and reviewed and edited the manuscript. MB-P investigated the data, wrote the original draft, and carried out the formal analysis. PA-R investigated the data, wrote the original draft, and reviewed and edited the manuscript. All authors contributed to the article and approved the submitted version.

## Conflict of Interest

The authors declare that the research was conducted in the absence of any commercial or financial relationships that could be construed as a potential conflict of interest.

## Appendix

**TABLE A1:** OECL-1 Scale (Ramos-Galarza et al., 2019b).

**Executive Function**	**Items**
EF 1: Error correction	IT 1. When an error is made because of your behavior in front of people around you, you look to solve it. IT 2. You correct the mistakes provoked by your behavior. IT 3. When doing an activity, you are able to correct the errors that keep you away from your proposed objective.
EF 2: Internal behavioral and cognition regulatory language	IT 4. When committing an error, your internal voice guides you to correct it. IT 5. When facing a task, your internal voice indicates what to do in order to achieve it successfully. IT 6. You have an internal voice that guides your actions.
EF 3: Limbic system conscious regulation	IT 7. You are able to react peacefully to different situations you encounter. IT 8. When angry, you are capable of calming down easily. IT 9. You are capable of managing your anger.
EF 4: Decision making	IT 10. You are capable of making decisions without difficulties, even in the face of easy and simple things. IT 11. You make decisions considering the implications of what results they could bring. IT 12. You have the capacity to make decisions independently.
EF 5: Future consideration of the consequences of actions	IT 13. You are capable of anticipating the consequences of your actions. IT 14. When you make a decision in your life, you consider the consequences of it. IT 15. When you are doing an activity, you are capable of noticing the consequences it will bring.
EF 6: Goal-directed behavior	IT 16. Your daily behavior aims at the achievement of a future plan. IT 17. Your daily activities aim to achieve an objective. IT 18. Your actions pursue a determined goal.
EF 7: Inhibitory control of automatic responses	IT 19. You allow others to talk without interrupting. IT 20. You think before saying things to other people. IT 21. You act reflectively and not according to the first thing that crosses your mind.
EF 8: Creation of new behavioral repertoires	IT 22. You are able to modify your behavior to perform correctly in new places. IT 23. When finding yourself in a different place (country, city, community, etc.), you are capable of adapting successfully. IT 24. You have the capacity to create new behavioral repertoires to solve situations you never faced before.
EF 9: Cognitive-behavioral activity verification	IT 25. You check the writing and grammar of your text messages before sending them. IT 26. When finishing a meeting, you check to verify if your behavior was appropriate. IT 27. When you complete an evaluation, you check that all the questions have been answered before turning it in.

**TABLE A2:** Prefrontal Symptoms Inventory (Ruiz-Sánchez de León et al., 2012).

**Factor**	**Items**
Social behavior problems	1. I make inappropriate jokes in inappropriate situations. 2 I make comments about very personal matters in front of others. 3. I do or say embarrassing things. 4. I make inappropriate sexual comments.
Behavior management problems	5. I have difficulty starting an activity due to lack of initiative. 6. It is very hard to focus on something. 7. I cannot do two things at once, such as tidying up and talking on the phone. 8. I have trouble changing the subject during conversations. 9. I only do what I have to do when someone tells me. 10. I have trouble keeping up with a movie or book. 11. I can change from laughing to crying very easily. 12. I become very slow as if I am almost asleep. 13. It is hard to plan things in advance.
Emotional control problems	14. My emotions can change from happiness to sadness easily. 15. I get angry about meaningless things. I get angry easily. 16. I explode emotionally for no apparent reason.
